# Protein and muscle health during aging: benefits and concerns related to animal-based protein

**DOI:** 10.1093/af/vfz030

**Published:** 2019-09-28

**Authors:** Kyle J Hackney, Kara Trautman, Nathaniel Johnson, Ryan Mcgrath, Sherri Stastny

**Affiliations:** Department of Health, Nutrition, and Exercise Sciences, North Dakota State University, Fargo, ND

**Keywords:** aging, animal-based protein, muscle size, plant-based protein, sarcopenia

**Implications**

- Individuals aged at least 65 years are a fast-growing segment of the population.
- Age-related loss of muscle mass and strength will continue to have a significant economic impact unless dietary or exercise interventions are implemented.
- Increasing the ratio of animal-based protein relative to plant-based protein in the diet may help to mitigate age-related losses of muscle mass and strength.
- Animal-based protein sources, especially those that are lean or nutrient dense, are the most anabolic per gram.
- Additional health and environmental considerations are needed prior to increasing animal-based protein intake recommendations in the United States and globally.

## Introduction

**Figure 1.**
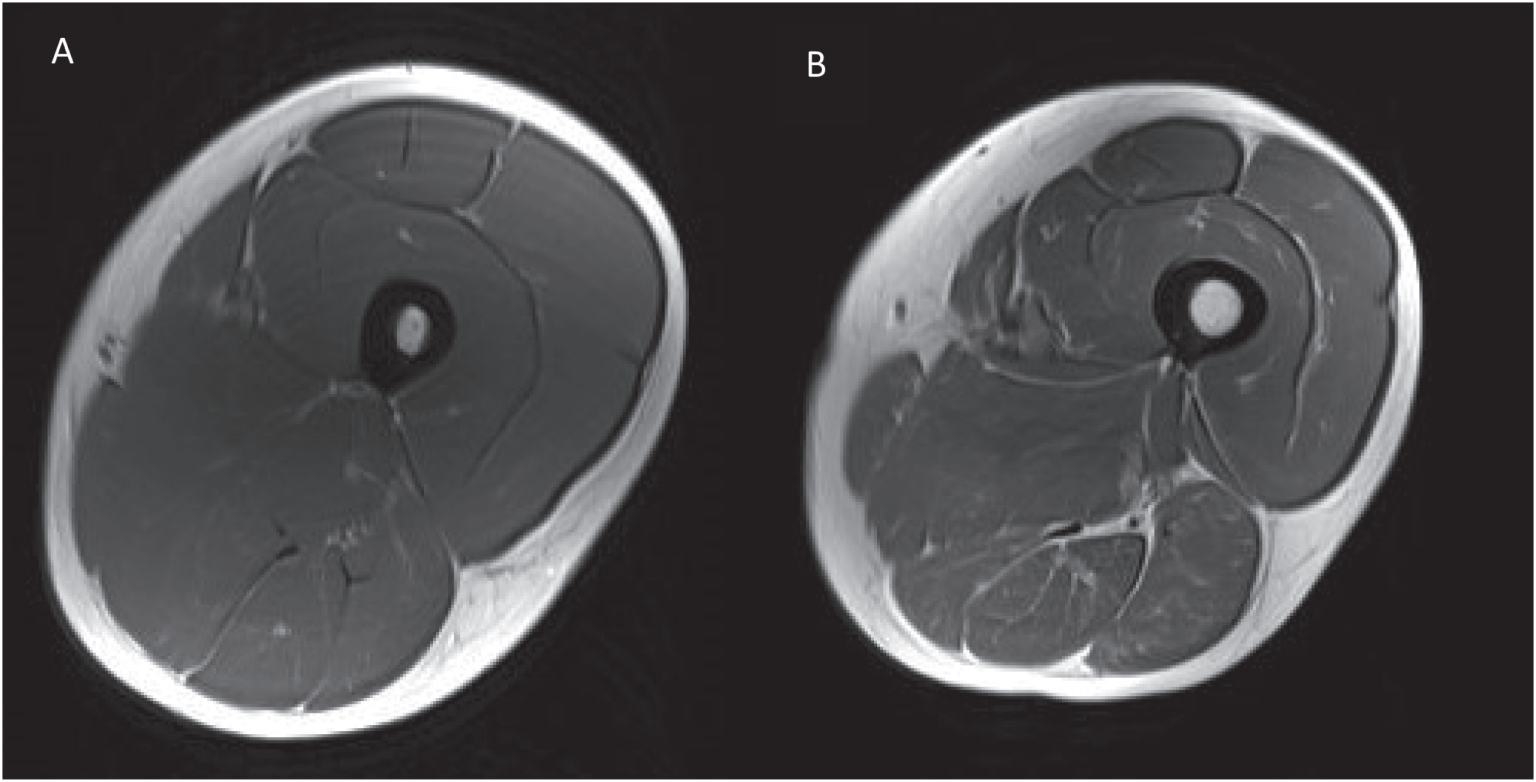
Representative MRI images of the upper leg showing muscle area (dark gray), inter/intramuscular adipose/fibrous tissue (white area within and between muscles), and subcutaneous adipose tissue (white area around all the muscle groups) in (A) younger, active and (B) older, inactive male. Muscle area is increased in the younger, active male by 55% compared with the older, active male (82.21 cm^2^ vs. 52.87 cm^2^), whereas lipid/fibrous area is increased 7× in the older, inactive male compared with the younger, active male (11.50 cm^2^ vs. 1.64 cm^2^).

It is estimated that by 2060, nearly one in four individuals in the United States (94 million) will be over the age of 65 (Vespa et al., 2018). A well-known consequence of aging is the loss of skeletal muscle mass and strength, which are often referred to as sarcopenia and dynapenia, respectively (Clark and Manini, 2008). Collectively, sarcopenia and dynapenia can alter energy metabolism and lead to the loss of physical function, ultimately manifesting as functional disability. Skeletal muscle wasting specifically, occurs at a rate of 1% to 2% per year beginning at age 50 yr and may significantly affect up to 45% of older adults (Baumgartner et al., 1998). For example, Figure 1 shows a magnetic resonance imaging axial scan of the left upper leg muscles in (Figure 1A) a younger, active male and (Figure 1B) an older, inactive male. When analyzed via color thresholding for various tissue types and concentrations, notice the dramatic decrease in muscular area (dark gray color) in the inactive, older adult, which is accompanied by an increase in lipid infiltration and fibrous tissue (white color) within the muscle area relative to the active, younger adult. Although this is not the same individual, it is recognized that nearly all humans will have a similar profile over time unless an intervention (physical activity, exercise, dietary, pharmacological) occurs to slow the progression of skeletal muscle loss and adipose tissue infiltration. As a result, sarcopenia may lead to a greater economic burden related to treatments associated with loss of muscle mass and physical function (Janssen et al., 2004).

Skeletal muscle mass is governed by the daily balance between the rates of muscle protein synthesis and muscle protein degradation. Shifting the balance toward protein synthesis results in the addition of muscle protein, whereas shifting the balance toward protein degradation results in the loss of muscle protein (Phillips et al., 2009). Vigorous physical activity and exercise act as stimuli for muscle protein synthesis, which can last for several days following each bout (MacDougall et al., 1995), especially if the intensity is high enough to trigger anabolic hormone production (e.g., growth hormone, insulin-like growth factor 1) and cell-signaling cascades within muscle tissue. Food intake, in particular, dietary protein, is also a potent stimulator for muscle protein synthesis; however, it is more short-lived lasting only 4 to 5 h following ingestion (Fujita et al., 2007). The essential amino acids, especially leucine, appear to be the primary triggers for the initiation of muscle protein synthesis by way of mammalian target of rapamycin complex (mTORC1) phosphorylation and downstream markers that increase translation efficiency (Drummond et al., 2009).

**Figure 2.**
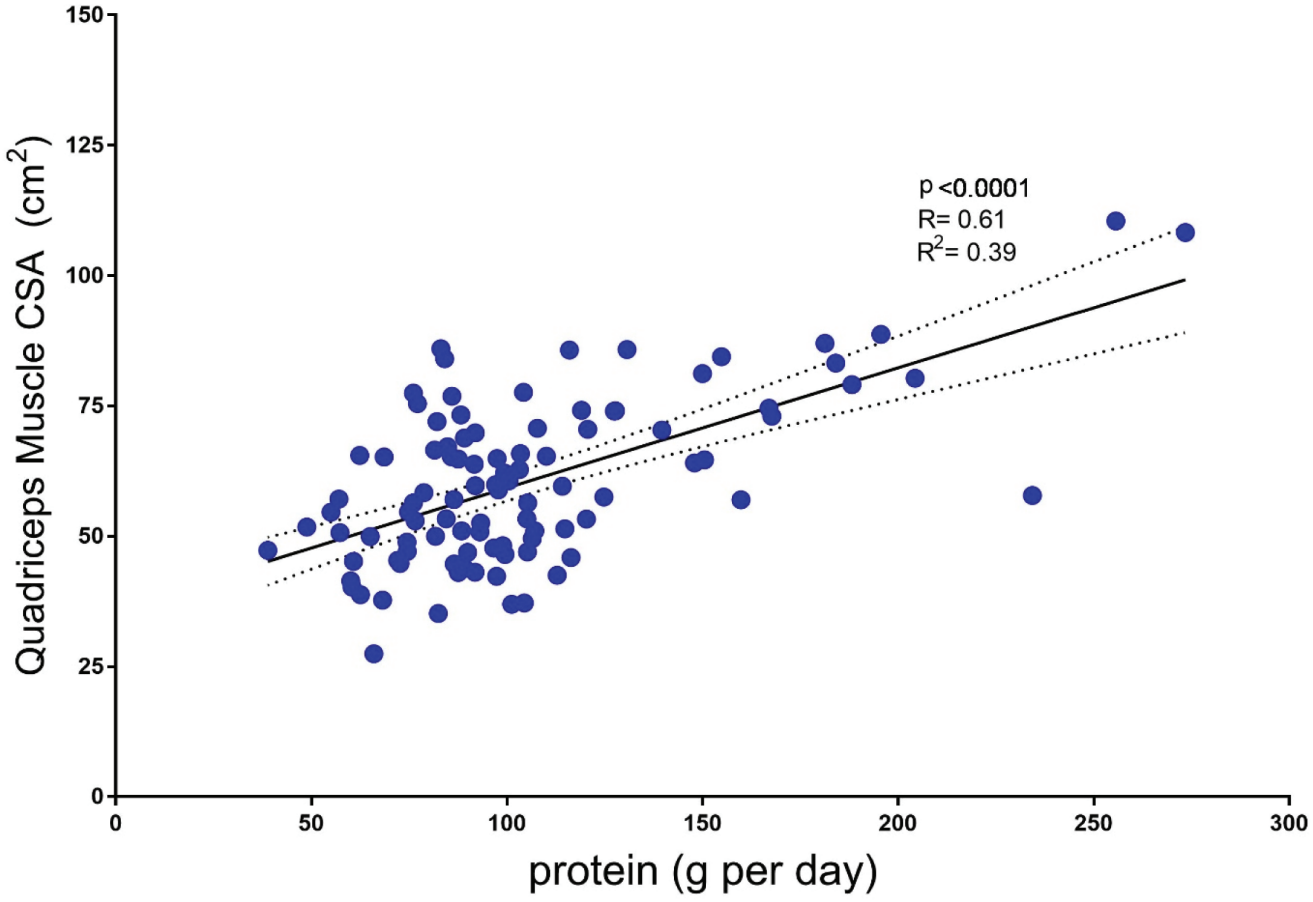
Association between total protein intake and muscle cross-sectional area of the quadriceps muscle group in healthy younger and middle-aged adults.

Given this mechanistic evidence, elevated protein intake has been extensively studied to help preserve age-related muscle mass loss. For example, the recommended daily allowance for protein, or the daily amount sufficient to meet nutrition requirements for 97% to 98% of the population is 0.8 g per kg body mass (Institute of Medicine, 2005). To increase muscle health during the aging process, others have recommended 1.0 to 1.6 g per kg body mass, which is nearly a doubling for the current recommended daily allowance (Deutz et al., 2014). Older adults, especially women, often do not meet the recommended daily allowance for total protein. There are many reasons why protein intake could go down during aging. These include but are not limited to low appetite, dentition and other medical conditions, functional limitations that limit shopping, lack of knowledge on food preparation, and food insecurity (Deutz et al., 2014). Data from our Muscle, Metabolism, and Ergogenic workgroup suggest a significant and positive association with total protein intake from the diet and muscle cross-sectional area of the quadriceps muscles in younger and middle-aged adults (Figure 2). Therefore, providing specific recommendations that highlight protein type and quality is an important first step for providing aging adults with a protein intake level that emphasizes optimal consumption, instead of minimal intake, for muscle health.

Total protein intake from the diet or supplementation is categorized into two basic types: 1) plant-based protein sources (e.g., soy, rice, pea, oat, wheat, rice, legumes, beans, nuts) and 2) animal-based protein sources (e.g., meat, whey, poultry, fish, eggs, and milk). Adults aged 51 to 70 yr in the United States consume about 65% of protein from animal sources and approximately 35% from plant-based sources (Berner et al., 2013). Most (20%) of this animal-based protein was from red meat (14% beef and remainder pork, lamb, game, or beef/pork combined with other meats), 18% from dairy (including milk and cheese with less than 2% yogurt), 14% from poultry (95% chicken), and the remainder distributed among seafood, fish, and eggs. Accordingly, beef, pork, and chicken represent the majority of animal-based protein consumed in the United States. The more recent NHANES data (2015 to 2016) reveal increased consumption of poultry (47 g/wk), decreased consumption of unprocessed red meat (from 340 g/wk 1999 to 2000 to 284 g/wk 2015 to 2016), and no change in consumption patterns for processed red meats (182 g/wk; Zeng et al., 2019). van Vliet et al. (2015) recently provided an excellent critical review in relation to animal- and plant-based protein in relation to anabolic potential in skeletal muscle. In brief, Protein Digestibility Amino Acid Scores for milk, whey, egg, soy protein isolate, and casein are 1.000; soy and beef ranged from 0.91 to 0.92; and whole wheat, oat, and pea ranged from 0.45 to 0.67 (van Vliet et al., 2015). Thus, some animal and plant proteins were reported to have similar anabolic potential. However, there are limitations with the Protein Digestibility Amino Acid Scores (index may be replaced by Digestible Indispensable Amino Acid Score; Wolfe et al., 2016) given anabolic potential in skeletal muscle may vary based on essential amino acid content, digestibility, and absorption (Phillips, 2012). For instance, when stimulation of post-prandial muscle protein synthesis has been evaluated by stable isotope tracers methodology in combination with resistance exercise, whey (Yang et al., 2012), milk (Wilkinson et al., 2007), and beef (Phillips, 2012) appear to be superior to soy proteins, which suggests beneficial prediction of muscle mass with animal-based protein sources. Thus, animal-based proteins appear more anabolic than a similar dose of plant proteins.

**Figure 3.**
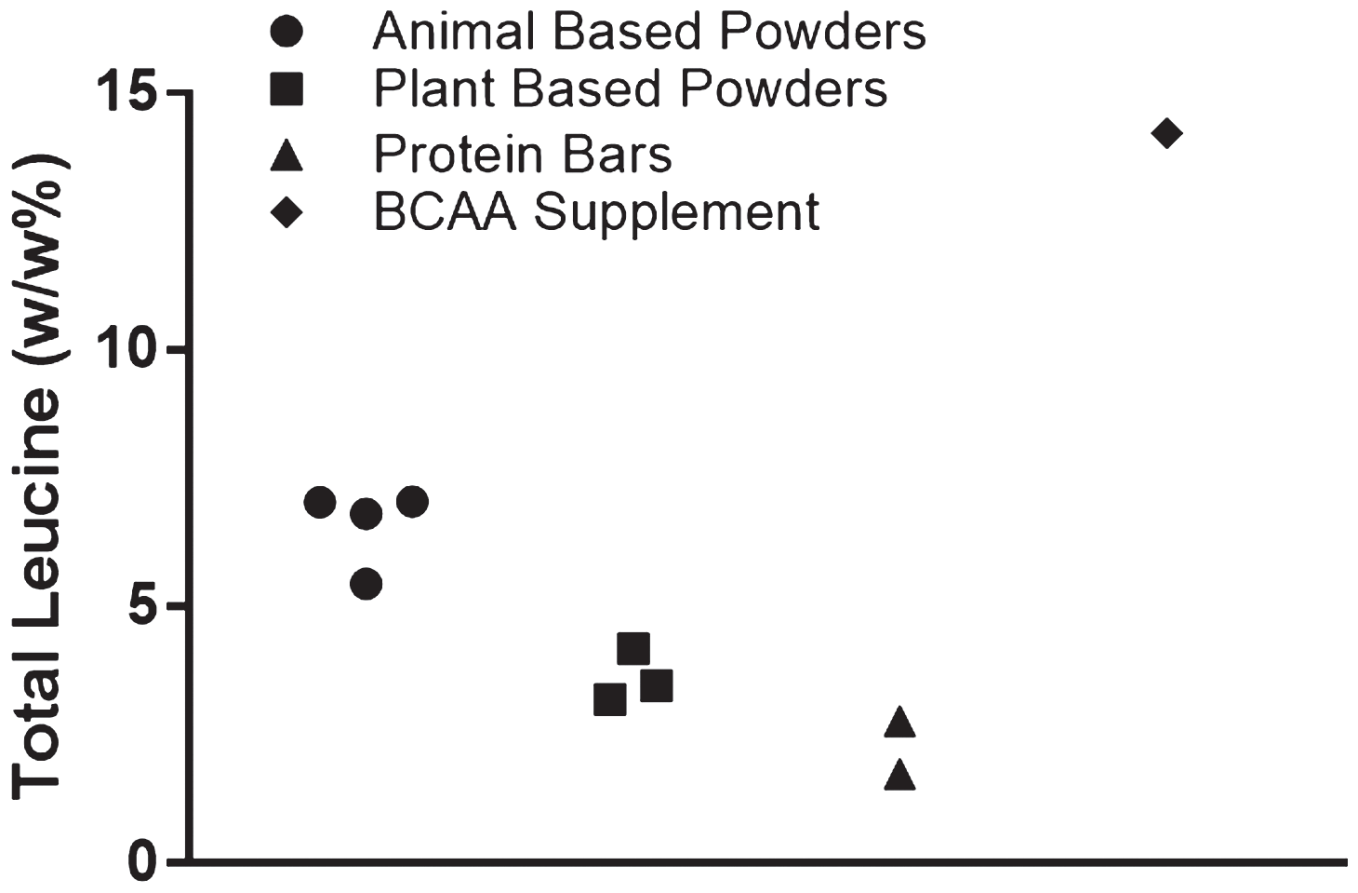
Leucine content evaluated in various protein sources relative to weight (w).

There is additional cross-sectional evidence that indicates protein intake from animal sources are associated with increased muscle mass with aging. Lord et al. (2007) found animal-based protein intake was an independent predictor of an index of muscle mass (*fat-free mass*/*height squared*) in women >60 yr of age (Lord et al., 2007). Higher beef intake was also significantly associated with greater appendicular muscle mass index in nonobese males ≥50 yr of age (Morris and Jacques, 2013). Furthermore, leg lean mass was higher for participants in the highest quartiles of total protein and animal-based protein compared with those in the lowest quartiles (Sahni et al., 2015). Animal-based proteins generally are greater sources of lysine, leucine, and methionine relative to plant-based sources; thus, even larger amounts of plant-based protein are needed to have a similar influence on muscle size compared with animal-based proteins (Figure 3).

## Key Nutrients Associated with Animal-Based Protein

Lean beef is particularly interesting for older adults. There are 29 cuts of beef that meet the labeling requirements for “lean” or “extra lean” (5 g or less fat and 2 g or less of saturated fat). A lean 3 ounce serving of beef, 6 cups of cooked brown rice, and 1 scoop of whey-protein all contribute about 2.15 g of leucine, whereas a 1/2 cup of almonds or soybeans has about 0.4 g of leucine. A 3-ounce portion of lean meat also provides about 10% of recommended daily calories, 37% of vitamin B12, 33% of zinc, 25% of niacin, plus over 10% of recommended iron, riboflavin, and other nutrients. Beef is, therefore, an example of a nutrient-rich food, important for those limiting or limited in their daily consumption of total calories. Iron, although not a nutrient of value for older adults, is instead very important as a nutrient of interest (shortfall nutrient) for premenopausal females, children, and during pregnancy. Meat foods provide heme iron, which is more bioavailable than nonheme plant-based iron. There is a high prevalence of vitamin B12 deficiency among older adults. A deficiency may be associated with confusion and other conditions that hurt quality of life (Stover, 2010).

## Animal-Based Protein Sources and General Health

Although there is general support for increasing animal-based protein intake from the diet for preserving muscle mass during aging, animal-based protein consumption has recently become a highly debated topic in scientific literature and popular media. Animal-based proteins may be associated with chronic diseases such as heart disease, colorectal cancer, breast cancers, prostate cancers, and bone demineralization (Cho et al., 2006; Kaluza et al., 2015; Isanejad et al., 2017; Diallo et al., 2018). However, additional studies published conflicting results, which further muddle our understanding on how animal-based proteins are related to human health needs (Hannan et al., 2000; Lin et al., 2004; Sanjoaquin et al., 2004; Alexander et al., 2009). It is likely that a person can live a reasonably healthy, low-risk life while consuming meat. To do so, one must consider that the mechanisms of the diseases and conditions listed above are multifaceted and that nutrition is only a single facet. For example, the high-fat content in animal-based proteins has been linked to cancers and cardiovascular disease incidence, but some of the fats found in lean meats and low-fat dairy (e.g., monounsaturated fatty acids) have shown to be beneficial (Lin et al., 2004; Diallo et al., 2018). Further investigation has suggested that carbohydrate intake may be a larger contributor in these conditions even more than saturated fats (Barclay et al., 2008).

Fat content is not the only concern that researchers have with meat. The difference between processed meat and unprocessed meat in regard to disease risk has been highly debated as increased nitrates, phosphate-containing preservatives, hydrocarbons, and sulfates are potential carcinogens (Lin et al., 2004; Cho et al., 2006; Diallo et al., 2018). In one study, women who consumed more than three servings per week of red meat as processed meats were 2.3 times more likely to develop hormone-positive breast cancers than women consuming less than one serving per month (Cho et al., 2006). Another study showed that consuming >50 g of processed meat per day was associated with a 23% increase in the risk of developing heart failure (Kaluza et al., 2015). However, it does not seem that processed meats are the only concerns as unprocessed meat has also been associated with a 23% higher risk of stroke (Bernstein et al., 2012). In addition, it does not seem that red meats are the only animal-based product that have been related to chronic illness. Egg intake of more than two eggs per week has been associated with breast and prostate cancers through hormonal mechanisms related to insulin-like growth factor 1, dioxine, and/or choline (Wu et al., 2016; Marcondes et al., 2019). Although these risks are concerning upon first glance, comparisons between vegetarians and their counterparts have reported no significant differences in the associations of colorectal cancer, fat free mass, fat mass, muscle size, muscle strength, phosphocreatine, creatine, or resting energy expenditure (Haub et al., 2002; Sanjoaquin et al., 2004). Protein has also been beneficial in bone maintenance regardless of hypotheses regarding potential renal acid load due to high sulfates leading to decreased urinary calcium through bone calcium buffering (Hannan et al., 2000; Isanejad et al., 2017). Although others have shown total protein intakes well above the recommended daily allowance of 0.8 g/kg/day have been associated with lower femoral neck, lumbar spine, and total bone mineral density and bone mineral content (Isanejad et al., 2017). Overall, though there are risks associated with eating larger amounts of animal-based protein, they are less risky than using tobacco, drinking alcohol, and consuming large amounts of white bread (Sanjoaquin et al., 2004). Thus, the decision to incorporate animal-based products is a personal choice but can be done so safely by consuming a diet rich in fruits and vegetables and swapping out fried and/or processed foods for healthy, low-fat, calcium-containing alternatives (e.g., cottage cheese or yogurt for bacon at breakfast; Heaney and Layman, 2008; Astrup et al., 2011). In addition, increasing physical activity is associated with the reduction in cardiovascular disease (Lear et al., 2017) and various cancers (Moore et al., 2016) and therefore should be incorporated within a diet composed of higher animal-based proteins.

## Animal-Based Protein and the Environment

Another concern related to elevating the promotion and intake of animal-based proteins is the effect that their production has on the environment and whether elevated production is sustainable. Animal-based proteins require a greater amount of energy, water, and land than plant-based proteins (Pimentel and Pimentel, 2003). For example, it has been estimated that on average 6 kg of plant protein is needed to produce 1 kg of animal protein (Pimentel and Pimentel, 2003). Similarly, others have projected that 1 kg of beef protein requires five times more water to produce compared with 1 kg of cereal protein (Mekonnen and Hoekstra, 2012). The increased demands of producing animal proteins can lead to nutrient depletion of soil (Menzi et al., 2010) and environmental pollution (Steinfeld et al., 2006). Additionally, the production of animal proteins is related to the emission of greenhouse gasses (Lesschen et al., 2011), and this problem is exasperated by increased deforestation for food production (Steinfeld et al., 2006). It is estimated that a shift from a Western diet, characterized by high intake of animal proteins, is a more sustainable dietary practice for reducing greenhouse gas emissions and land use by 70%, and water use by 50% (Aleksandrowicz et al., 2016). For more detailed discussion, Grossi et al. (2019) recently published a review that addresses the effects that livestock production has on the environment.

## Conclusions

The loss of muscle mass and strength within the rapidly growing aging population is an emerging public health epidemic. Increase in dietary protein intake above the recommended daily allowance is recommended for skeletal muscle preservation, but the ratio of animal-based proteins to plant-based proteins selected by the consumer is an individual choice filled with various ethical, economical, and environmental decisions. Regardless of protein selection, physical activity participation is also recommended in combination with increased fruit and vegetable intake as part of a balanced diet to not only enhance muscle health, but also increase longevity.

## About the Authors

**Kyle J. Hackney** is an Associate Professor at North Dakota State University, Fargo, North Dakota, within the Department of Health, Nutrition, and Exercise Sciences. He earned a BA degree from Hope College, Holland, Michigan; an MEd from Wayne State University, Detroit, MI; and a PhD from Syracuse University, Syracuse, NY. He leads the Muscle, Metabolism, and Ergogenics Work team, which is a group of students and faculty dedicated to 1) understanding how exercise, nutrition, and environmental stimuli interact to produce phenotype changes (e.g., skeletal hypertrophy, strength gain, fatigue resistance) and 2) implementing these regimens across a variety populations that may benefit (e.g., recreational exercisers, elite athletes, aging adults, individuals with disabilities, or other specialized populations).

**Kara Trautman** is a Doctoral Candidate at North Dakota State University, Fargo, ND, in the Department of Health, Nutrition, and Exercise Sciences. She completed her MS at the University of Central Missouri, Warrensburg, MO. The overarching aims of her research are to 1) further understand the role of nutrition and exercise on skeletal muscle health (particularly regarding hypertrophy, strength, and muscle quality); 2) implement findings to populations at risk for loss of lean mass and strength due to disuse and atrophy (i.e. astronauts and aging or injured individuals); and 3) implement findings to the community and young researchers to increase the knowledge of healthy aging.

**Nathaniel Johnson** is a Doctoral Student at North Dakota State University in the Department of Health, Nutrition, and Exercise Sciences. He has a Bachelor of Arts in Nutrition from St. John’s University, Collegeville, MN. He is interested in the mechanisms of skeletal muscle hypertrophy and how they can be modulated through diet and exercise to maximize health.

**Ryan McGrath** is an Assistant Professor in the Department of Health, Nutrition, and Exercise Sciences at North Dakota State University. He earned a BA in Physical Education from the University of Jamestown, Jamestown, ND; MS in Kinesiology from the University of North Dakota, Grand Forks, ND; and PhD with an Exercise Science Emphasis from the University of Idaho, Moscow, ID. His research interests include topics related to physical activity and health in older adults and persons with disabilities.

**Sherri Nordstrom Stastny** is a Professor in the Department of Health, Nutrition, and Exercise Sciences, North Dakota State University, Fargo, ND. She is a Board Certified Specialist in Sports Dietetics with the Academy of Nutrition and Dietetics. She is a member of the dietetics faculty and has developed and led research studies and classes in Nutrition for Human Performance and all aspects of foods and foodservice management since 2002. She is a gerontologist and has an overall interest in healthy aging and well-being of individuals across the lifespan.
